# Gene expression profile of CD14^+^ blood monocytes following lifestyle-induced weight loss in individuals with metabolic syndrome

**DOI:** 10.1038/s41598-020-74973-2

**Published:** 2020-10-20

**Authors:** Ronald Biemann, Kirsten Roomp, Fozia Noor, Shruthi Krishnan, Zhen Li, Khurrum Shahzad, Katrin Borucki, Claus Luley, Jochen G. Schneider, Berend Isermann

**Affiliations:** 1 Institute of Laboratory Medicine, Clinical Chemistry and Molecular Diagnostics, University of Leipzig, Leipzig, Germany; 2 Luxembourg Centre for Systems Biomedicine (LCSB), University of Luxembourg, Luxembourg, Luxembourg; 3 Institute of Clinical Chemistry and Pathobiochemistry, Otto-von-Guericke University, Magdeburg, Germany; 4 Department of Internal Medicine II, Saarland University Medical Center at Homburg/Saar, Homburg, Germany

**Keywords:** Diseases, Endocrine system and metabolic diseases, Metabolic syndrome

## Abstract

Lifestyle-induced weight loss is regarded as an efficient therapy to reverse metabolic syndrome (MetS) and to prevent disease progression. The objective of this study was to investigate whether lifestyle-induced weight loss modulates gene expression in circulating monocytes. We analyzed and compared gene expression in monocytes (CD14^+^ cells) and subcutaneous adipose tissue biopsies by unbiased mRNA profiling. Samples were obtained before and after diet-induced weight loss in well-defined male individuals in a prospective controlled clinical trial (ICTRP Trial Number: U1111-1158-3672). The BMI declined significantly (− 12.6%) in the treatment arm (N = 39) during the 6-month weight loss intervention. This was associated with a significant reduction in hsCRP (− 45.84%) and circulating CD14^**+**^ cells (− 21.0%). Four genes were differentially expressed (DEG’s) in CD14^+^ cells following weight loss (ZRANB1, RNF25, RB1CC1 and KMT2C). Comparative analyses of paired CD14^+^ monocytes and subcutaneous adipose tissue samples before and after weight loss did not identify common genes differentially regulated in both sample types. Lifestyle-induced weight loss is associated with specific changes in gene expression in circulating CD14^+^ monocytes, which may affect ubiquitination, histone methylation and autophagy.

## Introduction

Metabolic syndrome (MetS) represents a cluster of risk factors for cardiovascular disease (CVD) and type 2 diabetes mellitus. Experimental and observational evidence suggests that obesity-associated inflammation plays a central role in metabolic dysfunction and disease progression. Adipose tissue is primarily composed of adipocytes and immune cells such as macrophages. It has been increasingly recognized as a major source of circulating proinflammatory cytokines, which are typically observed in obesity-associated metabolic dysfunction^1^. Following prolonged caloric overload and adipocyte hypertrophy, IL-6 and other proinflammatory molecules such as MCP-1 (monocyte chemoattractant protein-1) are secreted by adipocytes themselves^2^. Obesity-associated adipose tissue inflammation promotes the accumulation of adipose tissue macrophages (ATMs), which may be derived either from peripheral blood monocytes or from local macrophage proliferation^3^. ATMs interacting with hypertrophic adipocytes promote insulin resistance and inflammation of adipose tissue. ATM frequency and proinflammatory gene expression in adipose tissue are positively associated with adipocyte size and negatively associated with weight loss in obesity^4,5^.

A possible source of proinflammatory ATMs is peripheral circulating monocytes. Monocyte frequency is elevated in overweight and obese individuals compared with lean individuals^6^. Moreover, elevated monocyte frequency is associated with an increased risk for cardiovascular disease^7^. Experimental models imply that obesity-associated metabolic stress promotes the “priming of circulating monocytes” i.e., hypersensitization of blood monocytes to chemokines, resulting in increased recruitment of monocyte-derived macrophages into the sites of inflammation^8,9^. Thus, obesity-associated changes in peripheral blood monocytes may promote the progression of adipose tissue inflammation and aggravate obesity-associated metabolic dysfunction.

Lifestyle-induced weight loss significantly improves metabolic and cardiovascular outcomes in patients with MetS^10^. This suggests that not only the frequency but also the gene expression profile of peripheral blood monocytes may change upon weight loss. Our study addresses the question of whether lifestyle-induced weight loss modulates the gene expression of peripheral circulating blood monocytes in individuals with MetS who are at high risk for type 2 diabetes mellitus and CVD. We analyzed and compared the gene expression profiles of paired CD14^+^ monocyte samples and corresponding adipose tissue samples before and after lifestyle-induced weight loss in well-defined individuals with MetS in a prospective controlled clinical trial (ICTRP Trial Number: U1111-1158-3672).

## Results

### Clinical and laboratory measurements

Before and after the 6-month period, clinical parameters and body composition were determined in participants of both arms. The analyzed study population did not differ in the distribution of age, sex or parameters of MetS at baseline (Table 1, Supplementary Table S3).

**Table 1:** Clinical parameters of individuals with MetS before and after the 6-month treatment period.

	Treatment arm		Control arm	
	Before	6 month	Before	6 month
**Age**				
Median	48.0		46.5	
IQR	(45.0–51.0)		(42.5–48.0)	
**BMI**				
Median	33.6	28.8***	33.4	34.14***
IQR	(31.2–35.6)	(26.5–31.6)	(32.4–35.4)	(33.1–35.5)
**Body weight (kg)**				
Median	108	93.5***	103	104*
IQR	(99.0–117)	(84.5–106)	(96.8–109)	(98.7–110)
**Triglycerides (mmol/l)**				
Median	1.90	1.26***	2.02	2.26*
IQR	(1.39–2.85)	(0.95–1.8)	(1.57–3.19)	(1.87–3.61)
**Total cholesterol (mmol/l)**				
Median	6.08	5.20**	5.94	6.05
IQR	(4.85–6.69)	(4.33–6.25)	(5.17–7.13)	(4.78–7.08)
**HDL-cholesterol (mmol/l)**				
Median	1.21	1.45***	1.28	1.29
IQR	(1.05–1.41)	(1.22–1.61)	(1.10–1.63)	(1.05–1.59)
**LDL-cholesterol (mmol/l)**				
Median	3.50	3.18*	3.57	3.40
IQR	(2.71–4.35)	(2.40–4.01)	(2.76–4.81)	(2.52–4.14)
**Free fatty acids (mmol/l)**				
Median	0.55	0.50	0.63	0.53
IQR	(0.42–0.71)	(0.35–0.59)	(0.51–0.90)	(0.41–0.80)
**HOMA-IR**				
Median	3.13	1.29***	2.10	2.11*
IQR	(1.89–5.00)	(0.91–2.40)	(1.55–3.10)	(1.71–3.45)
**Total fat mass (kg)**				
Median	31.9	23.8***	26.1	28.9*
IQR	(25.9–36.7)	(20.5–29.3)	(23.9–35.4)	(23.8–36.3)
**Leptin (ng/ml)**				
Median	12.5	5.57***	10.1	12.8***
IQR	(8.06–17.2)	(2.82–8.75)	(7.55–19.9)	(7.85–18.4)
**IL-6 (pg/ml)**				
Median	2.70	2.20*	1.85	2.05
IQR	(1.90–4.00)	(1.90–2.80)	(1.50–2.57)	(1.50–4.00)
**hsCRP (mg/l)**				
Median	3.40	1.10***	3.70	2.20
IQR	(1.30–6.60)	(0.60–3.60)	(1.25–5.87)	(1.27–4.82)
**WBC (Gpt/l)**				
Median	6.25	6.20	6.70	6.95
IQR	(5.30–7.70)	(4.70–7.30)	(5.97–7.65)	(6.13–7.77)
**CD14**^**+**^** cell frequencies (10**^**7**^**/20 ml)**				
Median	1.10	0.80*	1.29	1.10
IQR	(0.80–1.40)	(0.50–1.40)	(0.98–1.70)	(1.00–1.37)

Data are presented as the median (interquartile range, IQR).

*BMI* Body Mass Index, *HDL* high-density lipoprotein cholesterol, *LDL* low-density lipoprotein cholesterol, *HOMA-IR* homeostasis model of assessment index, *IL-6* interleukin-6, *hsCRP* high sensitive C-reactive protein, *WBC* white blood cell count.

The Wilcoxon Signed-Rank test was used to analyze differences in paired samples of the treatment arm (N = 39) and the control arm (N = 12), *p < 0.05, **p < 0.01, ***p < 0.001. No differences were found between both arms with regard to age and BMI before weight loss (Mann–Whitney U test).

Participants in the treatment arm reduced their individual BMI by 12.6%. Other significantly changed parameters included decreased serum levels of triglycerides (− 30.5%), LDL cholesterol (− 11.9%), HOMA-IR (− 48.7%), leptin (− 62.6%), IL-6 (− 4.7%) and hsCRP (− 45.4%).

Following the 6-month observational period, participants of the control arm significantly increased BMI (1.8%) and total body fat mass (5.2%). No other significant changes were observed.

In both arms, no progression to overt type 2 diabetes was observed during the 6-month period. In general, the changes in these clinical and laboratory measurements are comparable with those obtained in the whole cohort that initially completed the study^11^.

### CD14^+^ cells

Although lifestyle-induced weight loss had no effect on total leukocyte levels, the frequency of CD14^**+**^ cells was significantly decreased (− 21.0%). The purity of CD14^**+**^ cells, as quantified by FACS, was between 99.0 and 99.4% (Table 2), indicating that the obtained results are not confounded by CD14^**−**^ blood cells.

**Table 2:** Quality control of CD14^+^ samples and total RNA obtained.

	Treatment arm		Control arm	
	Before	6 month	Before	6 month
**CD14**^**+**^**, CD3**^**–**^** FACS (%)**				
Median	99.2	99.0	99.4	99.3
IQR	(98.6–99.5)	(98.6–99.4)	(99.2–98.7)	(98.4–99.4)
**RNA QC (RIN)**				
Median	9.70	9.90	9.40	9.75
IQR	(9.60–9.80)	(9.60–10.0)	(9.20–9.70)	(9.50–9.87)

The RNA integrity number (RIN) gives the quality of the extracted RNA. Data are presented as the median (interquartile range, IQR).

**Figure 1:**
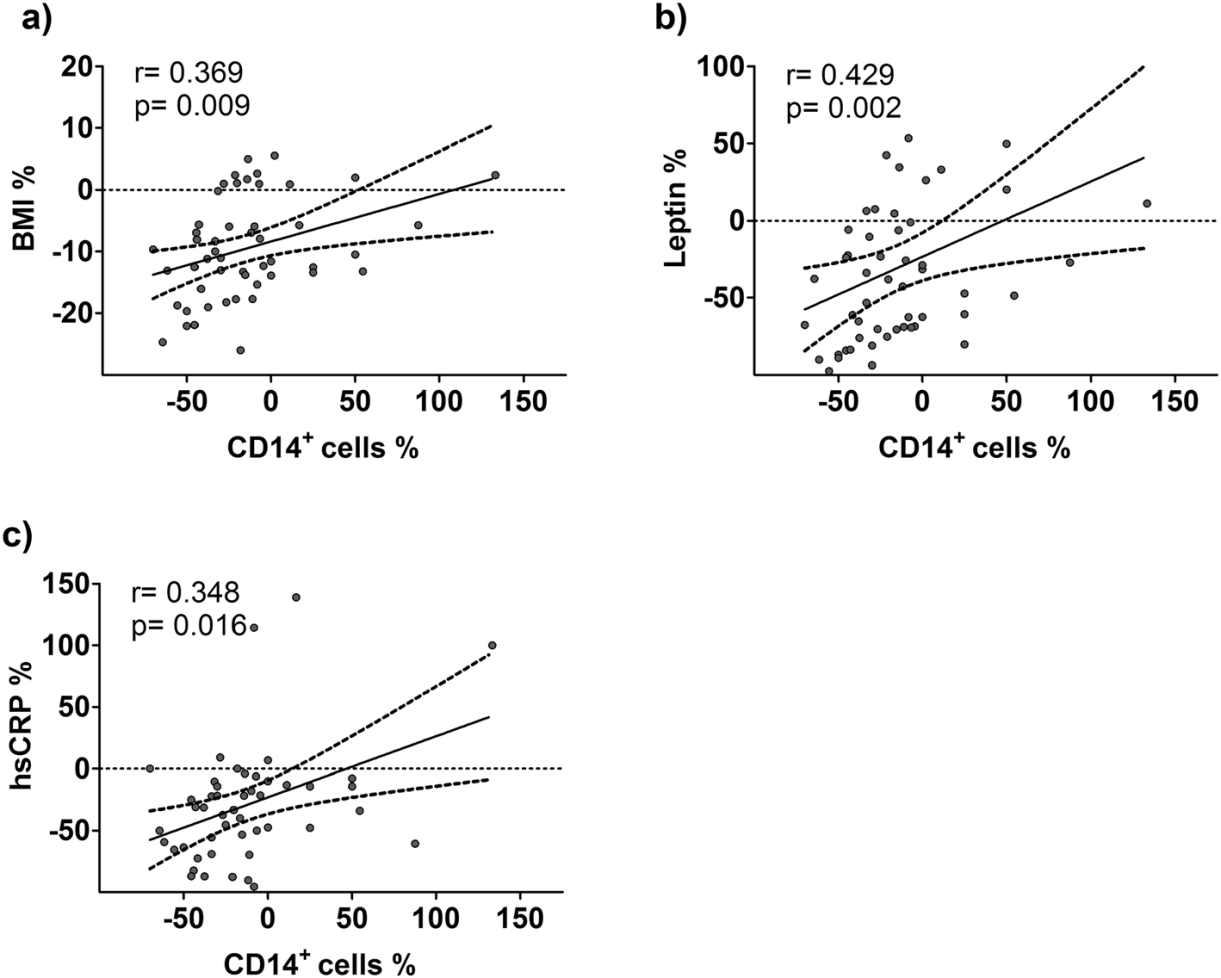
Relationship between changes in CD14^+^ cell frequencies and clinical or laboratory parameters. Spearman correlation (2-tailed) was used to analyze the correlation between relative changes in CD14^+^ cell frequencies and BMI (**a**), leptin (**b**) or hsCRP (**c**) in participants of both arms. Data are shown as individual data points. The regression line is given as the mean ± 95% confidence interval.

To assess the relationship between CD14^**+**^ cell frequencies and clinical or laboratory parameters characteristic of MetS, we determined bivariate correlations (Supplementary Table S1). The relative changes in CD14^**+**^ cell frequencies were positively correlated with BMI (r = 0.37; *p* < 0.009), leptin (r = 0.43; *p* < 0.002) and hsCRP (r = 0.35; *p* < 0.016) (Fig. 1, Supplementary Fig. S1).

### Gene expression analysis

To identify differentially expressed genes in CD14^**+**^ cells following lifestyle-induced weight loss, gene expression was analyzed by microarray analysis. Raw data were normalized to compensate for systematic variation (Supplementary Fig. S2). After normalization, we calculated a median coefficient of variation (CV) of 4.08% across all cells in the experiment (78 samples × 53,617 cells). All samples were included for differential gene expression analysis.

**Figure 2:**
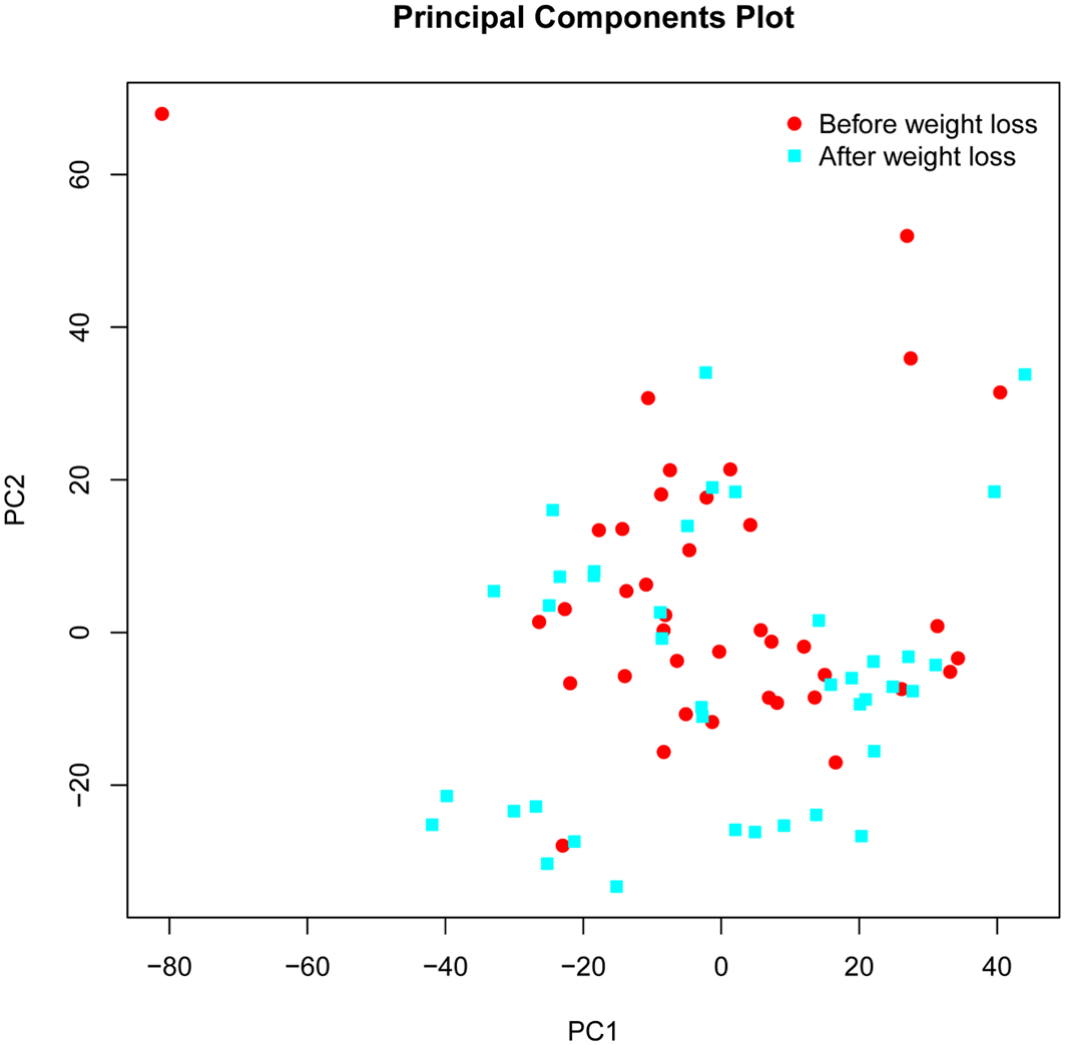
Principal component analysis. The principal component analysis (PCA) plot was used to visualize the variance in gene expression data in participants of the treatment arm at baseline (red symbols) and after the 6-month intervention period (blue symbols). The first principal component (PC1) on the x-axis is the linear combination comprising the largest percentage of variation in the dataset. The second principal component (PC2), located on the y-axis, captures the second largest percentage of variance. PCA was generated using the affycoretools package in R (R Core Team, 2018, R: A Language and Environment for Statistical Computing. R Foundation for Statistical Computing, Vienna. https://www.R-project.org).

PCA was used to visualize the variance of the gene expression data in the weight loss group. The first principal component (PC1) on the x-axis accounts for most of the variation in the dataset. The second principal component (PC2), located on the y-axis, captures the second largest percentage of variance. Surprisingly, the PCA analysis did not clearly separate the two observational time points (treatment arm before and after the 6-month intervention period), indicating that gene expression in CD14^+^ cells remained largely unaffected despite weight loss (Fig. 2).

Differential gene expression was separately analyzed in paired CD14^+^ samples from the treatment and the control arm. The false discovery rate was controlled by correction for multiple testing using the Benjamini–Hochberg method. We identified four protein coding genes in paired CD14^+^ samples of the treatment arm that were differentially expressed after the 6-month intervention period (Table 3) namely, ZRANB1 (zinc finger RANBP2-type containing 1; adj. *p* = 0.006, log fold change = − 0.307), RNF25 (ring finger protein 25; adj. *p* = 0.049, log fold change = 0.24), RB1CC1 (adj. *p* = 0.049, log fold change = − 0.16) and KMT2C (Lysine Methyltransferase 2C; adj. *p* = 0.049, log fold change =  − 0.20).

**Table 3:** Differentially expressed genes in paired CD14^+^ samples and paired adipose tissue samples after the 6-month intervention period.

Gene name	Paired CD14^+^ samples: top 20 DEGs				Paired adipose tissue samples			
					Ranking of same 20 DEGs			
	Rank	logFC	aveExpr	adj.p.Val	Rank	logFC	aveExpr	adj.p.Val
ZRANB1	1	− 0.30	9.15	0.006	4216	− 0.19	9.05	0.404
RNF25	2	0.24	7.54	0.049	22,263	0.04	7.10	0.856
RB1CC1	3	− 0.16	9.21	0.049	23,485	− 0.04	8.25	0.870
KMT2C	4	− 0.20	10.36	0.049	898	− 0.27	8.19	0.146
MIR125A	5	0.17	3.93	0.060	11,422	− 0.08	3.34	0.663
EDEM1	6	− 0.22	8.68	0.070	15,779	− 0.04	6.76	0.757
ATP13A3	7	− 0.29	8.59	0.070	33,186	0.01	8.51	0.961
JMJD1C	8	− 0.24	10.47	0.070	10,763	− 0.10	8.93	0.647
SMEK1	9	− 0.16	9.31	0.070	2797	− 0.11	7.57	0.318
EIF3J	10	− 0.19	8.10	0.070	22,419	− 0.05	7.41	0.857
FBXO11	11	− 0.13	9.71	0.070	4751	− 0.12	8.84	0.434
CHD1	12	− 0.26	9.97	0.070	12,159	− 0.11	7.44	0.678
CCNT1	13	− 0.18	8.66	0.070	16,754	− 0.04	7.86	0.770
ARIH1	14	− 0.14	10.46	0.070	1907	− 0.15	9.72	0.246
USP9X	15	− 0.16	8.83	0.070	37,022	0.00	8.42	0.992
HGSNAT	16	− 0.15	9.54	0.082	8978	− 0.08	9.19	0.598
SESTD1	17	− 0.22	9.27	0.082	7018	− 0.12	9.83	0.538
TRPC6	18	0.15	4.27	0.086	7733	− 0.13	4.79	0.561
WDFY3	19	− 0.14	10.02	0.104	7355	− 0.09	8.94	0.551
BTAF1	20	− 0.14	9.87	0.106	294	− 0.23	8.05	0.066

The top 20 differential expressed genes (DEGs) in CD14^+^ samples are listed. Gene expression profiles were compared between CD14^+^ cells and corresponding subcutaneous adipose tissue samples (N = 36) and identified DEGs were ranked.

*LogFC* log2 fold change, *adj.p.Val* false discovery rate adjusted p-value.

**Figure 3:**
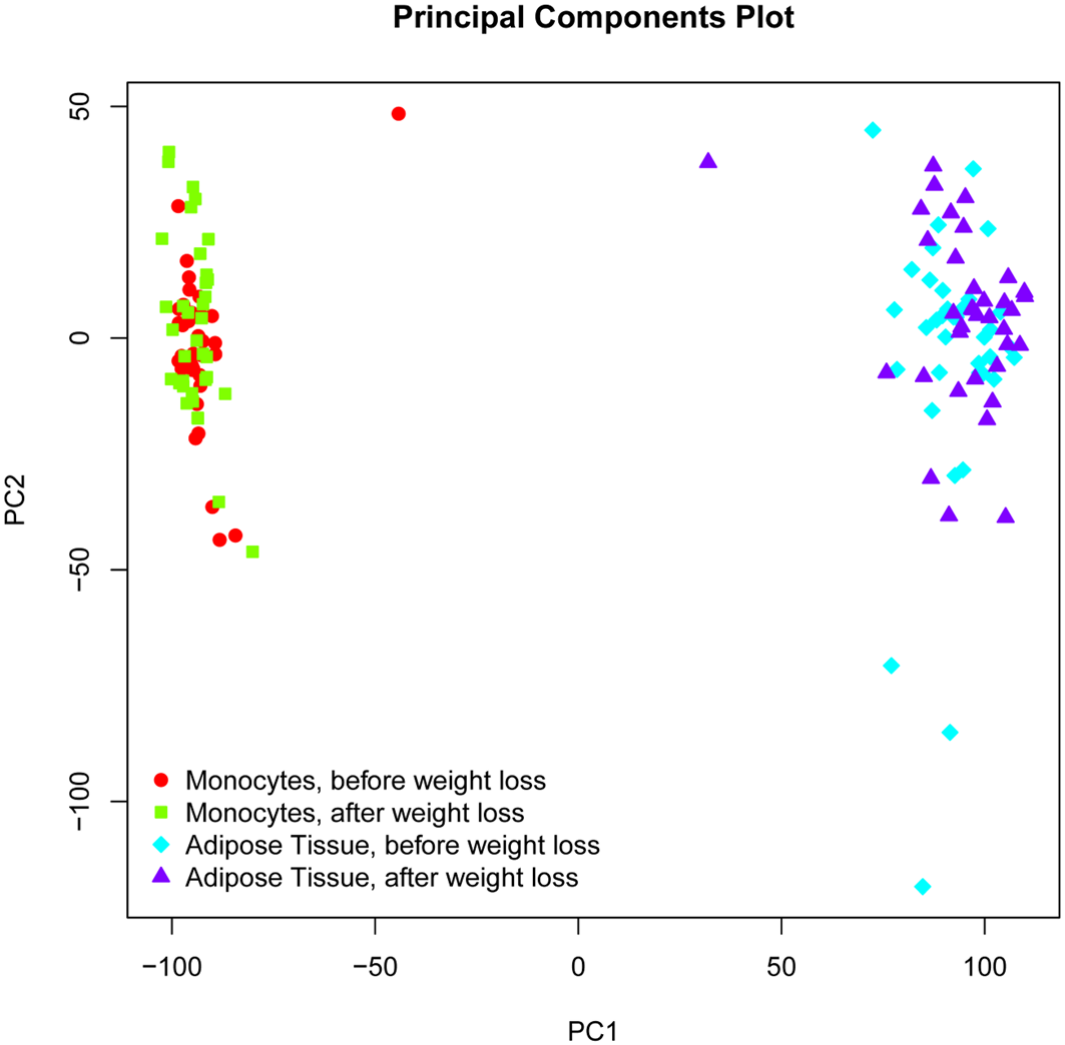
Principal component analysis of CD14^+^ and subcutaneous adipose tissue samples before and after weight loss. The principal component analysis (PCA) plot was used to visualize the variance in gene expression between corresponding CD14^+^ and subcutaneous adipose tissue samples each before (red, blue) and after (green, purple) weight loss (N = 36). The first principal component (PC1) on the x-axis is the linear combination comprising the largest percentage of variation in the dataset. The second principal component (PC2), located on the y-axis, captures the second largest percentage of variance. PCA was generated using the affycoretools package in R (R Core Team, 2018, R: A Language and Environment for Statistical Computing. R Foundation for Statistical Computing, Vienna. https://www.R-project.org).

Comparative analyses of gene expression of paired CD14^+^ monocyte samples and corresponding subcutaneous adipose tissue samples (N = 36) did not identify common genes differentially regulated in both sample types (Fig. 3, Table 3). Hence, the altered gene expression observed for the four genes was specific for CD14^+^ cells.

### Subgroup analysis in individuals with a large change in hsCRP

**Figure 4:**
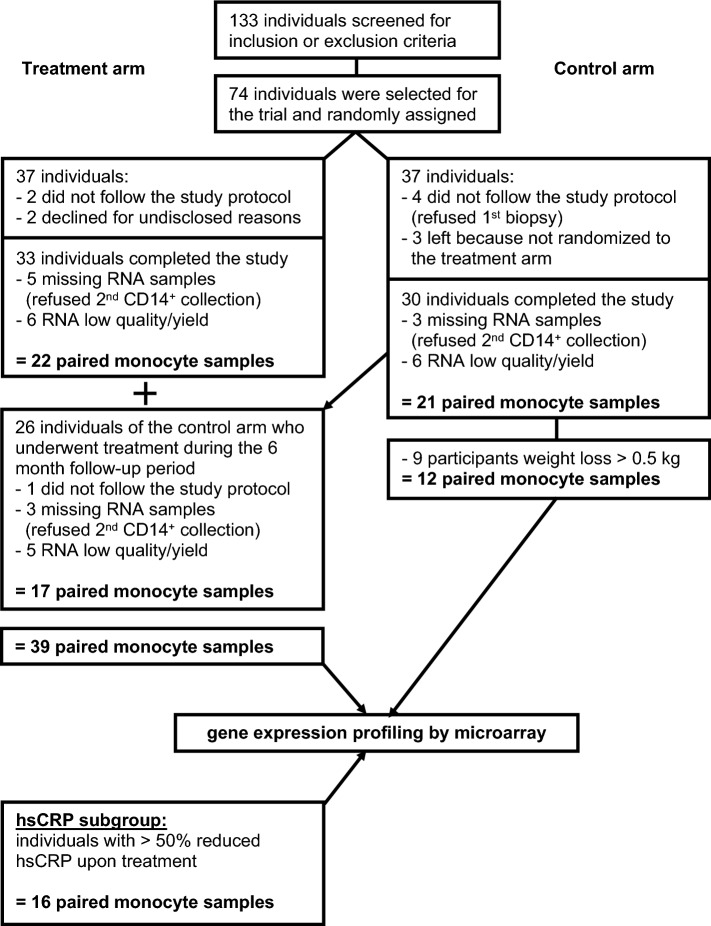
Schematic study design. The study is embedded in a two-armed, controlled, monocentric, randomized, 6-month intervention trial. Paired blood samples were collected before and after the 6-months intervention period. Individuals of the control arm were invited during the follow-up period to participate subsequently in the treatment arm. Samples from participants of the control arm who lost more than 0.5 kg of weight during the study period and samples with low RNA quality were excluded from analysis. In total, 39 samples (treatment arm) and 12 samples (control arm) were used for unbiased gene expression profiling of purified CD14^+^ monocyte samples. Within the treatment arm, a subgroup characterized by an at least 50% reduction of elevated hsCRP following weight loss was analyzed (N = 16, hsCRP subgroup).

As the reduced frequency of peripheral CD14^**+**^ cells was positively correlated with hsCRP, we speculated this may reflect an improvement in obesity-associated low-grade inflammation upon weight loss. Thus the altered gene expression may be more pronounced in individuals with a significant reduction in hsCRP. To this end, we performed a subgroup analysis, including only individuals from the treatment arm, in which the elevated hsCRP levels declined by at least 50% (N = 16, hsCRP subgroup, Fig. 4). Clinical and laboratory parameters of the hsCRP subgroup are shown in Supplementary Table S2. Weight loss and clinical parameters were comparable between the hsCRP subgroup and the entire treatment arm except for inflammatory markers hsCPR and IL-6, which were more strongly reduced in the hsCRP subgroup (see Table 1, Supplementary Table S2).

However, PCA analysis did not show clear separation when comparing gene expression before and after weight loss in the hsCRP subgroup (Supplementary Fig. S3). Furthermore, we did not observe significant gene expression alterations following correction for multiple testing.

## Discussion

Recent studies suggest that obesity-associated inflammatory mediators drive functional adaptations of circulating peripheral blood monocytes towards a proinflammatory phenotype^12–14^. According to the expert panel of the AHA/ACC/TOS, lifestyle-induced weight loss reduces the risk of developing type 2 diabetes mellitus and CVD^10^. Hence, we hypothesized that lifestyle-induced weight loss may modulate gene expression in peripheral blood monocytes and that genes regulated in monocytes upon weight loss could serve as potential biomarkers or even therapeutic targets. This hypothesis was tested using an unbiased approach in a well-characterized and well-matched cohort.

Following lifestyle-induced weight loss we identified four differentially expressed genes in CD14^+^ monocytes (ZRANB1, RNF25, RB1CC1 and KMT2C). The downregulated gene ZRANB1, which is also known as Trabid (TRAF-binding protein domain), encodes the K29- and K33-specific deubiquitinase ZRANB1^15^. ZRANB1 has been shown to regulate the Wnt signaling pathway^16^ and to play a role in the regulation of cell morphology and cytoskeletal organization^17^. Moreover ZRANB1 deficiency in macrophages and dendritic cells impairs the induction of IL-12 and IL-23 by the regulation of histone modifications at the IL-12 promoter, indicating a role of ZRANB1 in mediating inflammatory responses^18^ and macrophage activation^19^.

The upregulated RNF25 encodes a ubiquitin E3 ligase that has been identified as an interacting protein with the transactivation domain of the p65 subunit of NF-κB^20^. Hence RNF25 may promote NF-κB-induced inflammation. Moreover, its RING finger motif has been shown to bind to ubiquitin-conjugating enzymes and to promote E2-dependent ubiquitination^21^.

The upregulated gene RB1CC1 encodes the RB1-inducible coiled-coil protein 1, which is known to promote autophagosome formation through direct interaction with Atg16L1^22^.

In addition, we observed a downregulation of KMT2C, which encodes the histone methyltransferase Lysine *N*-methyltransferase 2C that regulates gene transcription by mediating mono- and tri-methylation of histone H3 at lysine 4^23^.

Taken together identified genes regulate ubiquitination, autophagy and histone methylation in CD14^+^ monocytes. Our results indicate that ubiquitination and autophagosome formation might be increased following lifestyle-induced weight loss. Autophagy is a cellular degradation pathway which maintains cellular homeostasis through degradation and recycling of cellular proteins in response to environmental conditions. It has been shown that insulin inhibits the activation of autophagy by mTOR (mechanistic target of rapamycin)^24^. Hence, we hypothesize that weight loss associated insulin reduction may cause observed induction of autophagy and ubiquitination in CD14^+^ monocytes. The net-effect on monocyte function induced by the altered expression of these four genes, which regulate fundamental pathways related to ubiquitination, histone methylation and autophagy, needs to be analyzed in detail in future studies.

Serum levels of adipose tissue-related proinflammatory cytokines correlate strongly with CRP, which is predominantly produced in hepatocytes but also by local macrophages, endothelial cells or adipocytes upon IL-6 stimulation^25^. CRP has been recognized as a relevant prognostic risk factor for cardiovascular disease, especially in association with abdominal obesity^26^. In the present study, plasma levels of IL-6 and CRP were increased before the intervention, reflecting obesity-associated chronic low-grade inflammation at baseline, and significantly declined following lifestyle-induced weight loss. The reduction in inflammatory markers is in line with other studies that investigated the effect of weight loss by lifestyle changes or by surgical interventions. Overall, weight loss has been associated with a linear decline in CRP levels^27^. Accordingly, we observed a strong correlation between changes in hsCRP levels and BMI during the 6-month intervention.

Together with elevated proinflammatory mediators, increased levels of monocyte frequencies have been reported in overweight and obese compared with lean individuals^6^. An increased frequency of monocytes has been proposed to aggravate obesity-associated low-grade inflammation^28^. In our study, we observed a decline in peripheral CD14^+^ cell frequencies following lifestyle-induced weight loss. Participants of the treatment arm were instructed to increase physical activity, to reduce calorie intake and to perform a low-carbohydrate diet with preference for low-GI carbohydrates. Moreover, participants in the treatment arm recorded daily body weight and received weekly written feedback commenting on their individual weight progress. Beyond these instructions, no special diet, e.g. specific macronutrients, was recommended. Hence, it is unfortunately not possible to separate the effects of the different components of the 6-month-long telemonitored lifestyle-induced weight loss program on CD14^+^ cell frequencies. However, the decline in CD14^+^ cells correlated moderately with parameters that reflect weight loss and inflammatory response, such as BMI, hsCRP and leptin. These observations suggest that lifestyle-induced weight loss is sufficient to reduce obesity-associated inflammation and peripheral blood monocyte frequencies.

Considering that monocyte gene expression might be driven by inflammatory signals, we performed a hsCRP subgroup analysis including monocytes from individuals in which the baseline hsCRP was reduced by at least 50%. This analysis did not reveal genes that were differentially expressed following lifestyle-induced weight loss. Considering the markedly reduced body weight and the unaltered gene expression profile in the hsCRP subgroup analysis, we propose that altered gene expression in peripheral blood monocytes is independent of weight loss-associated attenuation of chronic low-grade inflammation. However, this subgroup analysis included fewer samples (N = 16) than the calculated sample size (N = 34), which is a major limitation.

Obesity is associated with increased accumulation of ATMs from around 5% in lean up to 50% in obese individuals^5^. Migration of peripheral blood monocytes into adipose tissue has been proposed as a major source of ATMs. While the current study cannot directly evaluate this hypothesis, comparative analyses of gene expression of paired CD14^+^ monocytes and subcutaneous adipose tissue samples before and after weight loss did not identify common genes differentially regulated in both sample types. Our results support the notion that changes observed in CD14^+^ cells were specific for this cell population and that macrophages in the adipose tissue respond differently at the gene-expression level upon weight loss.

Recent studies demonstrated that the majority of tissue resident macrophages originate from embryonic progenitor cells^29^. In addition, preadipocytes have been shown to differentiate locally into macrophage-like cells that have phagocytic activity and that express macrophage-specific antigens such as F4/80, Mac-1, CD80, CD86, and CD45^30,31^. Indeed, it is now widely accepted that local processes are responsible for ATM accumulation in obesity^32^, a conclusion supported by our finding showing that weight loss is associated with specific gene expression alterations in peripheral CD14^+^ cells and that there is no overlap between CD14^+^ monocytes and adipose tissue gene expression upon weight loss.

The strengths of the current study include the prospective study design, a well-characterized study population with no differences between groups at baseline, a strong effect regarding weight loss as the primary outcome (12.6%) and robust statistical analyses of paired datasets (before and after intervention). In particular, the latter eliminates the changes in interindividual random differences as a source of accidental findings. All analyses were conducted in a blinded manner, and laboratory measurements were performed according to standard operating protocols, yielding high-quality data. Given the stringent selection criteria we only enrolled middle-aged Caucasian male participants with MetS for the study. This approach, while being a strength with respect to data validity, however precludes generalization of the data to e.g. to females, other racial/ethnic groups, older or younger individuals, or to individuals without MetS. A potential limitation of the study is our approach to increase sample size for differential gene expression analyses by adding participants of the original control arm, who participated in the subsequent follow-up treatment, to the treatment arm. However, baseline characteristics and weight loss were highly comparable between both groups, making this approach feasible. Moreover, our analysis is limited to gene expression. We did not address phenotypical alterations in circulating monocytes induced by posttranscriptional or posttranslational mechanisms, such as miRNA or receptor-ligand interactions. Furthermore, we cannot exclude changes in gene expression in subgroups of CD14^+^ monocytes.

In conclusion, lifestyle-induced weight loss is associated with altered gene expression in CD14^+^ peripheral blood monocytes, which may regulate ubiquitination, autophagy and epigenetic modifications.

## Methods

### Research design and study population

The study is embedded in a prospective, two-armed, controlled, monocentric, randomized, 6-month intervention trial aiming to identify changes in gene expression in individuals with MetS following lifestyle-induced weight loss. For this purpose, paired (i.e., before and after lifestyle-induced weight loss) blood samples and subcutaneous adipose tissue biopsies were collected at the Institute of Clinical Chemistry and Pathobiochemistry, Otto-von-Guericke University, Magdeburg, Germany. The trial was registered at the German Clinical Trials Register (ICTRP Trial Number: U1111-1158-3672)^11^.

The trial included nonsmoking, nondiabetic men aged between 45 and 55 years with MetS as defined by the National Cholesterol Education Program Adult Treatment Panel III guidelines: abdominal obesity (waist circumference > 102 cm or BMI > 30 kg/m^2^) combined with at least two of the following criteria: fasting triglyceride concentration ≥ 1.7 mmol/l; high-density lipoprotein (HDL) cholesterol < 1.05 mmol/l; fasting glucose ≥ 5.6 mmol/l; blood pressure ≥ 130/85 mmHg or treatment for hypertension. Exclusion criteria were smoking, type 2 diabetes mellitus, a history of surgical procedure for weight loss, severe renal dysfunction (creatinine concentration > 2.0 mg/dl), known liver disease, obesity of known endocrine origin or inability to walk at least 30 min per day. Out of 133 individuals who were recruited by an advertisement in a regional newspaper and screened for inclusion or exclusion criteria from May 2012 to August 2012, 74 individuals were selected for the trial. All participants underwent a structured education program about diet and the importance of physical activity. Individuals were randomly assigned to a 6-month-long telemonitored lifestyle-induced weight loss program (treatment arm, https://www.abcprogramm.de^33^) or a control arm as described previously^11^.

Participants of the treatment arm were advised to lower their calorie intake by 500 kcal/day and to perform a low-carbohydrate diet with preference for low-GI carbohydrates, as previously described^33^. Regarding exercise, participants were advised to increase their usual daily physical activity, but to keep the pulse below 120/min. Moreover, participants recorded daily body weight and received weekly written feedback commenting on their individual weight progress. This weight loss program was first shown to be effective in obese parents and obese children^34^.

At baseline and after 6 months, subcutaneous adipose tissue biopsies and peripheral blood samples were obtained from participants of both arms. All study participants were examined after 3 months using fasting blood glucose and glycated hemoglobin (HbA1c) to exclude new onset type 2 diabetes mellitus during the study.

Thirty participants in the control and 33 participants in the treatment arm completed the study^11^. For gene expression profiling, only paired sample sets with high RNA quality were selected, resulting in 22 paired monocytes samples in the treatment arm. During the follow-up, participants initially assigned to the control arm were offered enrollment in the treatment arm. 26 individuals participated in the subsequent 6-month weight loss program and received exactly the same weight loss intervention as participants of the initial treatment group (Supplementary Table S3). This yielded in 17 additional paired monocyte samples with high RNA quality. In the control arm, considering weight loss in participants as a possible confounding factor for data analysis, participants with weight loss of more than 0.5 kg during the study period were excluded from the analysis. Overall, this resulted in 39 and 12 paired sample sets from the treatment arm and the control arm, respectively, which were used for data analysis as shown in Fig. 4.

The gene expression profiles in paired CD14^+^ blood monocytes samples and corresponding subcutaneous adipose tissue biopsies (N = 36) were analyzed before and at the end of the 6-month intervention period in both arms.

### Clinical and laboratory measurements

Body weight and height were measured by qualified medical personnel according to standard operating protocols before and after 6 months. Body composition was analyzed by dual-energy X-ray absorptiometry in participants of the initial treatment group (N = 22) and the initial control group (N = 12). All blood samples were collected in the morning (8 am to 9 am) from the antecubital vein after a 12-h overnight fast. Glucose was determined in sodium fluoride plasma. Laboratory measurements were performed at the Institute of Clinical Chemistry and Pathobiochemistry, OvGU, Magdeburg, Germany, as described previously^11^. Concentrations of high sensitive CRP (hsCRP) were analyzed by a particle-enhanced immunoturbidimetric assay (Cobas c 501, Roche Diagnostics). Interleukin-6 was analyzed by a sandwich electrochemiluminescent immunoassay (ECLIA, Cobas e 601, Roche Diagnostics). Leptin concentrations were quantified using a commercially available enzyme linked immunosorbent assay (ELISA, according to the manufacturer’s instructions, BioVendor; Czech Republic) with an intraassay CV of 5.9%, interassay CV of 5.6% and a lower limit of detection of 0.2 ng/ml.

### CD14^+^ monocyte preparation

Peripheral blood mononuclear cells (PBMCs) were obtained from buffy coats of EDTA blood samples from overnight-fasted participants before and after 6 months. Twenty milliliters of whole blood was collected by standard venipuncture into blood collection tubes that contained K_2_EDTA. Each tube was diluted with two equal volumes of PBS. The diluted cell suspension was overlaid onto 15 ml of Ficoll-Paque and centrifuged (30 min; 400×*g*). The cell suspension was washed 3 times with PBS to remove platelets. To separate monocytes from lymphocytes, an additional iso-osmotic Percoll centrifugation was employed^35^. The monocyte phase was collected, and a positive selection of CD14^+^ cells was performed by adding MACS colloidal superparamagnetic microbeads (Miltenyi Biotec, Germany) conjugated with monoclonal anti-human CD14 in MACS buffer according to the manufacturer´s instructions. Briefly, after incubation of cells and microbeads (15 min at 4 °C), cells were washed with MACS buffer, resuspended, and loaded onto the separation column. Trapped CD14^+^ PBMCs were eluted, centrifuged, washed with PBS and stored in RNAlater (Sigma-Aldrich) at − 80 °C. A proportion of eluted cells was used to determine cell number and purity using flow cytometry. The purity was assessed by determining the number of cells that were positive for CD14 (Mouse Anti-Human CD14, BD, Catalog No. 555397) and negative for CD3 (Mouse Anti-Human CD3, eBioscience, Catalog No. 16-0037-85). The obtained CD14^+^ monocyte samples were sent to the Integrated Biobank of Luxembourg (IBBL) for storage at − 80 °C and RNA extraction.

### RNA quality assessment

RNA extraction and quality assessment were performed at the IBBL. Total RNA was extracted using the standard RNA extraction method with TRIzol (Invitrogen). The RNA concentration in each sample was assayed with an ND-1000 spectrophotometer (NanoDrop). RNA quality was assessed using an Agilent 2100 Bioanalyzer with an Agilent RNA 6000 nanokit (Agilent Technologies). The assessment of RNA quality was conducted to standardize the process of RNA integrity interpretation and to select samples that were suitable for downstream microarray analysis. The Bioanalyzer software algorithm calculates an RNA Integrity Number (RIN) on the basis of different regions of the entire electrophoretic trace of the RNA sample (pre-, 5S-, fast-, inter-, precursor-, post-region) and peaks (18S, 28S). Electropherograms are classified by a numeric system with 1 being the most degraded profile and 10 being the most intact. For microarray analysis, we selected only samples with RIN values above 8 (Table 2).

### Microarray analysis

Processing of RNA and transcriptomic profiling was performed at the European Molecular Biology Laboratory (EMBL). A total of 300 ng of total RNA was used to prepare labeled and purified sense-strand cDNA (Ambion WT Expression Kit). Reactions from all samples yielded sufficient cDNA for subsequent microarray analysis. Samples were hybridized to whole-transcript arrays (Affymetrix GeneChip Human Gene 2.0 ST Arrays) covering > 33,500 coding transcripts that include transcript variants and alternative splicing events. Hybridization was carried out according to the standard protocol provided by the manufacturer.

### Statistical analysis

The primary aim of this study was to characterize gene expression profiles of CD14^+^ monocytes before and after lifestyle-induced weight loss in well-defined individuals with MetS and to detect differentially expressed genes using microarray analysis. To calculate the required sample size the following sample size computational model was used: (https://bioinformatics.mdanderson.org/MicroarraySampleSize/MicroarraySampleSize.aspx). This algorithm takes normal distributed gene expression on log scale, false positive genes and independent gene measurements into consideration. We used GeneChip Human Gene 2.0 ST Arrays from Affymetrix which encode 40K RefSeq transcripts in this study. We applied 1 as the number of acceptable false positives in the experiment and selected a desired fold difference of 0.5. Our desired power is 0.95, which indicates that 95% of differentially expressed genes are likely to be detected by the experiment. We applied a level of 0.7 as a realistic value for standard deviation of the gene intensity measurement on the base-2 logarithmic scale for genes that are expressed at moderate to high levels^36^. This calculation results in a sample size per group of N = 34 for before and after weight loss.

Nonparametric tests were applied for all statistical analyses of clinical data. Data are given as the median and interquartile range (IQR). Differences between independent samples (treatment arm vs. control arm before or after 6 months, respectively) were analyzed by the Mann–Whitney U test. Paired samples were analyzed by the Wilcoxon Signed-Rank test. Correlations between relative changes during the 6-month intervention period were assessed by Spearman’s rank correlation. All calculations were performed using the IBM SPSS Statistics, version 22.0 (IBM Corporation, Armonk, NY, USA). The results were considered significant at *p* < 0.05.

### Gene expression analysis

Raw data were preprocessed with the Robust Multiarray Average algorithm (RMA; convolution background correction, quantile normalization and summarization based on the median polish algorithm, Supplementary Fig. S2)^37^. Differentially expressed genes were identified using the limma package in R^38^, which fits a linear model for each gene based on the given series of arrays, creates an appropriate contrast matrix to perform all pairwise comparisons, computes estimated coefficients and standard errors for a given set of contrasts and computes moderated t-statistics and log-odds of differential expression by empirical Bayes. Correction for multiple testing was performed using the Benjamini–Hochberg method to control the false discovery rate^39^. The principal components analysis (PCA) plot was generated using the affycoretools package in R^40^. The statistical procedure is a data compression method that allows for the transformation of a large set of possibly related variables into a new low-dimensional coordinate system that still contains most of the information.

### Ethics statement

This study was approved by the ethics committee at Otto-von-Guericke University, Magdeburg, Germany (No. 78/11). Written informed consent was obtained from all study participants. All human investigations were conducted according to the principles expressed in the Declaration of Helsinki.

## Supplementary information

Supplementary Information.

## References

[1] Kang YE (2016). The roles of adipokines, proinflammatory cytokines, and adipose tissue macrophages in obesity-associated insulin resistance in modest obesity and early metabolic dysfunction. PLoS ONE.

[2] Gustafson B, Hammarstedt A, Andersson CX, Smith U (2007). Inflamed adipose tissue: A culprit underlying the metabolic syndrome and atherosclerosis. Arterioscler. Thromb. Vasc. Biol..

[3] Zheng C (2016). Local proliferation initiates macrophage accumulation in adipose tissue during obesity. Cell Death Dis..

[4] Clément K (2004). Weight loss regulates inflammation-related genes in white adipose tissue of obese subjects. FASEB J..

[5] Weisberg SP (2003). Obesity is associated with macrophage accumulation in adipose tissue. J. Clin. Investig..

[6] Kullo IJ, Hensrud DD, Allison TG (2002). Comparison of numbers of circulating blood monocytes in men grouped by body mass index (<25, 25 to <30, or =30). Am. J. Cardiol..

[7] Waterhouse DF, Cahill RA, Sheehan F, McCreery C (2008). Prediction of calculated future cardiovascular disease by monocyte count in an asymptomatic population. Vasc. Health Risk Manage..

[8] Ullevig S (2012). NADPH oxidase 4 mediates monocyte priming and accelerated chemotaxis induced by metabolic stress. Arterioscler. Thromb. Vasc. Biol..

[9] Short JD (2017). Dyslipidemic diet-induced monocyte "priming" and dysfunction in non-human primates is triggered by elevated plasma cholesterol and accompanied by altered histone acetylation. Front. Immunol..

[10] Jensen MD (2014). 2013 AHA/ACC/TOS guideline for the management of overweight and obesity in adults: A report of the American College of Cardiology/American Heart Association Task Force on Practice Guidelines and the Obesity Society. J. Am. Coll. Cardiol..

[11] Biemann R (2016). Serum bile acids and GLP-1 decrease following telemetric induced weight loss: Results of a randomized controlled trial. Sci. Rep..

[12] Poitou C (2011). CD14dimCD16+ and CD14+CD16+ monocytes in obesity and during weight loss: Relationships with fat mass and subclinical atherosclerosis. Arterioscler. Thromb. Vasc. Biol..

[13] Gálvez I, Martín-Cordero L, Hinchado MD, Álvarez-Barrientos A, Ortega E (2019). Anti-inflammatory effect of β2 adrenergic stimulation on circulating monocytes with a pro-inflammatory state in high-fat diet-induced obesity. Brain Behav. Immunol..

[14] Chen X, Devaraj S (2014). Monocytes from metabolic syndrome subjects exhibit a proinflammatory M1 phenotype. Metab. Syndr. Related Disord..

[15] Licchesi JDF (2011). An ankyrin-repeat ubiquitin-binding domain determines TRABID's specificity for atypical ubiquitin chains. Nat. Struct. Mol. Biol..

[16] Tran H, Hamada F, Schwarz-Romond T, Bienz M (2008). Trabid, a new positive regulator of Wnt-induced transcription with preference for binding and cleaving K63-linked ubiquitin chains. Genes Dev..

[17] Bai SW (2011). Identification and characterization of a set of conserved and new regulators of cytoskeletal organization, cell morphology and migration. BMC Biol..

[18] Jin J (2016). Epigenetic regulation of the expression of Il12 and Il23 and autoimmune inflammation by the deubiquitinase Trabid. Nat. Immunol..

[19] Xing Z, Zganiacz A, Santosuosso M (2000). Role of IL-12 in macrophage activation during intracellular infection: IL-12 and mycobacteria synergistically release TNF-alpha and nitric oxide from macrophages via IFN-gamma induction. J. Leukoc. Biol..

[20] Asamitsu K, Tetsuka T, Kanazawa S, Okamoto T (2003). RING finger protein AO7 supports NF-kappaB-mediated transcription by interacting with the transactivation domain of the p65 subunit. J. Biol. Chem..

[21] Lorick KL (1999). RING fingers mediate ubiquitin-conjugating enzyme (E2)-dependent ubiquitination. Proc. Natl. Acad. Sci. U.S.A..

[22] Nishimura T (2013). FIP200 regulates targeting of Atg16L1 to the isolation membrane. EMBO Rep..

[23] Koemans TS (2017). Functional convergence of histone methyltransferases EHMT1 and KMT2C involved in intellectual disability and autism spectrum disorder. PLoS Genet..

[24] Kim J, Kundu M, Viollet B, Guan K-L (2011). AMPK and mTOR regulate autophagy through direct phosphorylation of Ulk1. Nat. Cell Biol..

[25] Calabro P, Chang DW, Willerson JT, Yeh ETH (2005). Release of C-reactive protein in response to inflammatory cytokines by human adipocytes: Linking obesity to vascular inflammation. J. Am. Coll. Cardiol..

[26] van Wijk DF (2016). C-Reactive protein identifies low-risk metabolically healthy obese persons: The European prospective investigation of cancer-norfolk prospective population study. J. Am. Heart Assoc..

[27] Selvin E, Paynter NP, Erlinger TP (2007). The effect of weight loss on C-reactive protein: A systematic review. Arch. Intern. Med..

[28] Kamei N (2006). Overexpression of monocyte chemoattractant protein-1 in adipose tissues causes macrophage recruitment and insulin resistance. J. Biol. Chem..

[29] Gomez Perdiguero E (2015). Tissue-resident macrophages originate from yolk-sac-derived erythro-myeloid progenitors. Nature.

[30] Charrière G (2003). Preadipocyte conversion to macrophage. Evidence of plasticity. J. Biol. Chem..

[31] Cousin B (1999). A role for preadipocytes as macrophage-like cells. FASEB J..

[32] Amano SU (2014). Local proliferation of macrophages contributes to obesity-associated adipose tissue inflammation. Cell Metab..

[33] Luley C (2014). Weight loss by telemonitoring of nutrition and physical activity in patients with metabolic syndrome for 1 year. J. Am. Coll. Nutr..

[34] Luley C (2010). Evaluation of three new strategies to fight obesity in families. J. Nutr. Metab..

[35] Seager Danciger J (2004). Method for large scale isolation, culture and cryopreservation of human monocytes suitable for chemotaxis, cellular adhesion assays, macrophage and dendritic cell differentiation. J. Immunol. Methods.

[36] Wei C, Li J, Bumgarner RE (2004). Sample size for detecting differentially expressed genes in microarray experiments. BMC Genomics.

[37] Irizarry RA (2003). Exploration, normalization, and summaries of high density oligonucleotide array probe level data. Biostatistics (Oxford, England).

[38] Ritchie ME (2015). limma powers differential expression analyses for RNA-sequencing and microarray studies. Nucleic Acids Res..

[39] Benjamini Y, Hochberg Y (1995). Controlling the false discovery rate: A practical and powerful approach to multiple testing. J. R. Stat. Soc. Ser. B (Methodological).

[40] MacDonald, J. W. Affycoretools: Functions useful for those doing repetitive analyses with Affymetrix GeneChips. R package version 1.60.1. (2018).

