# Supplementary material for: Gene expression profile of CD14+ blood monocytes following lifestyle-induced weight loss in individuals with metabolic syndrome
Source: Sci Rep. 2020 Oct 20;10:17855. doi: 10.1038/s41598-020-74973-2 (PMC7576128; doi:10.1038/s41598-020-74973-2)
Supplement: Supplementary file 1 — Supplementary Information. [file 41598_2020_74973_MOESM1_ESM.docx]

**Gene expression profile of CD14^+^ blood monocytes following lifestyle-induced weight loss in individuals with metabolic syndrome**

Ronald Biemann^1^ * & Kirsten Roomp^2^, Fozia Noor^2^, Shruthi Krishnan^1^, Zhen Li^1^, Khurrum Shahzad^1^, Katrin Borucki^3^, Claus Luley^3^, Jochen G. Schneider^2,4^ & Berend Isermann^1,^ ^3^

1 Institute of Laboratory Medicine, Clinical Chemistry and Molecular Diagnostics, University of Leipzig, Leipzig, Germany

2 Luxembourg Centre for Systems Biomedicine (LCSB), University of Luxembourg, Luxembourg, Luxembourg

3 Institute of Clinical Chemistry and Pathobiochemistry, Otto-von-Guericke University, Magdeburg, Germany

4 Department of Internal Medicine II, Saarland University Medical Center at Homburg/Saar, Homburg, Germany

**a)** **b)**


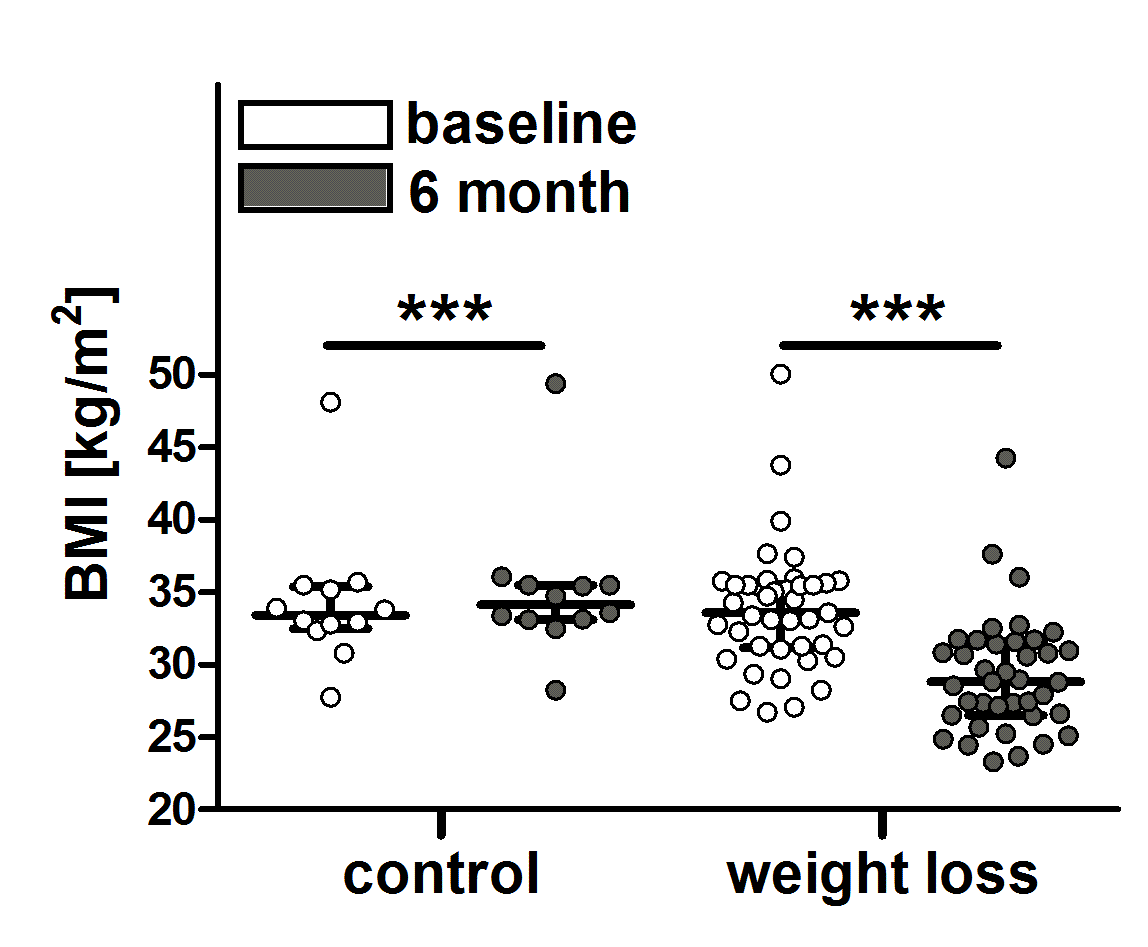

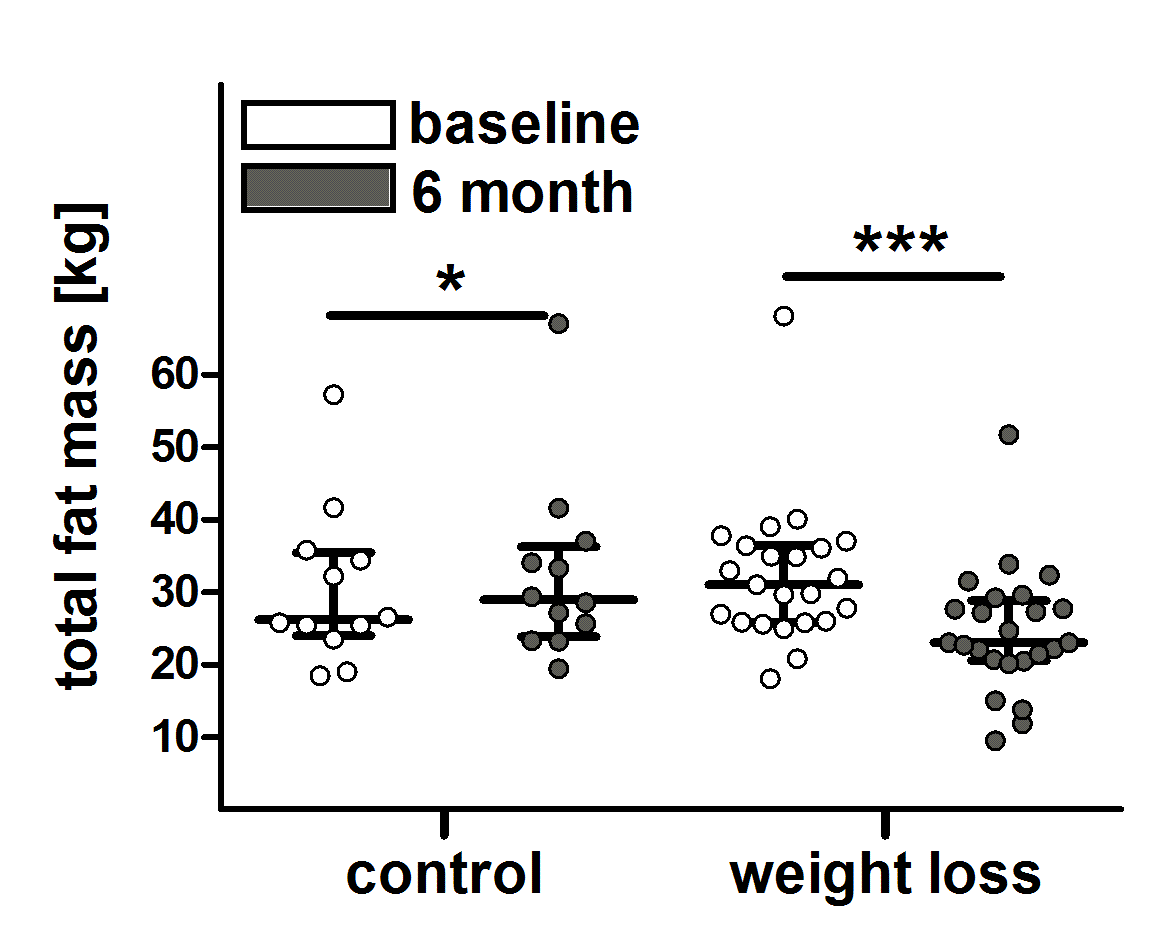


**c)** **d)**


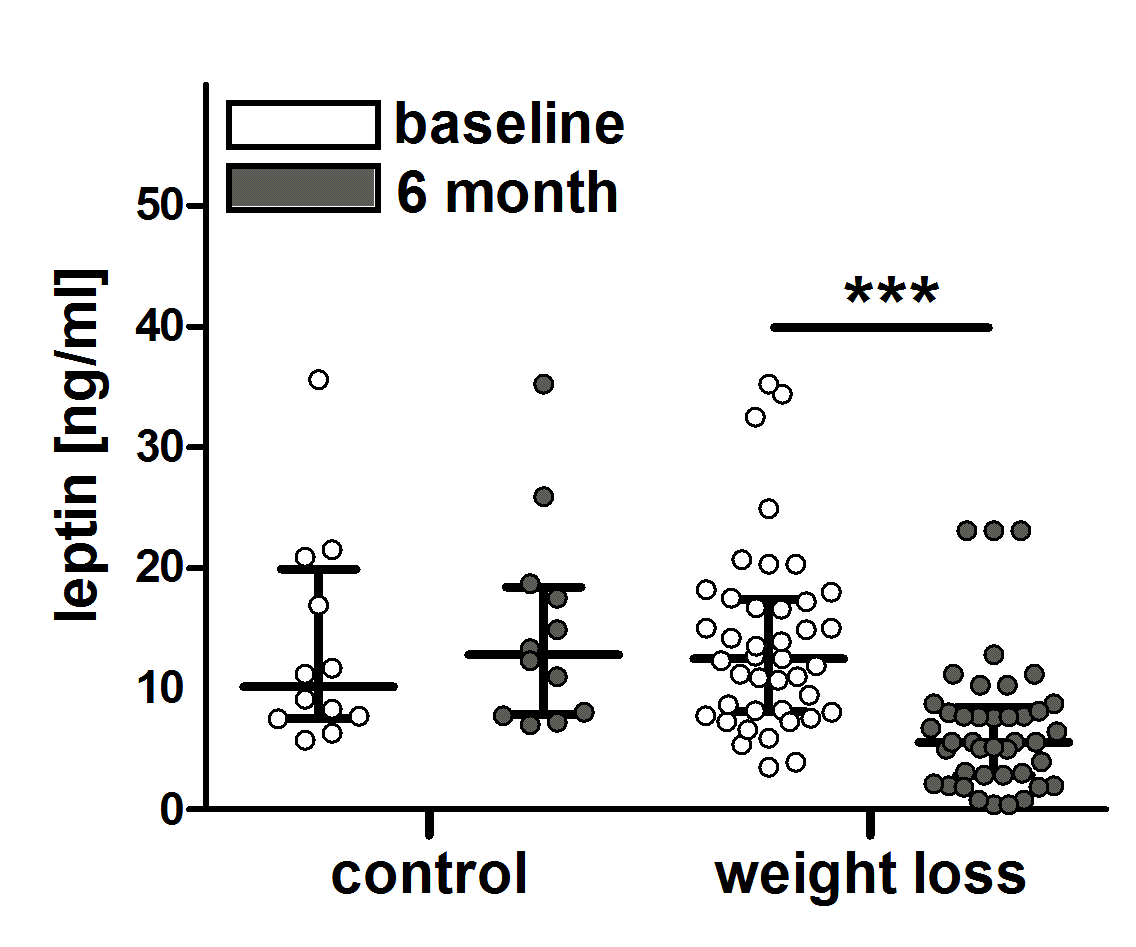

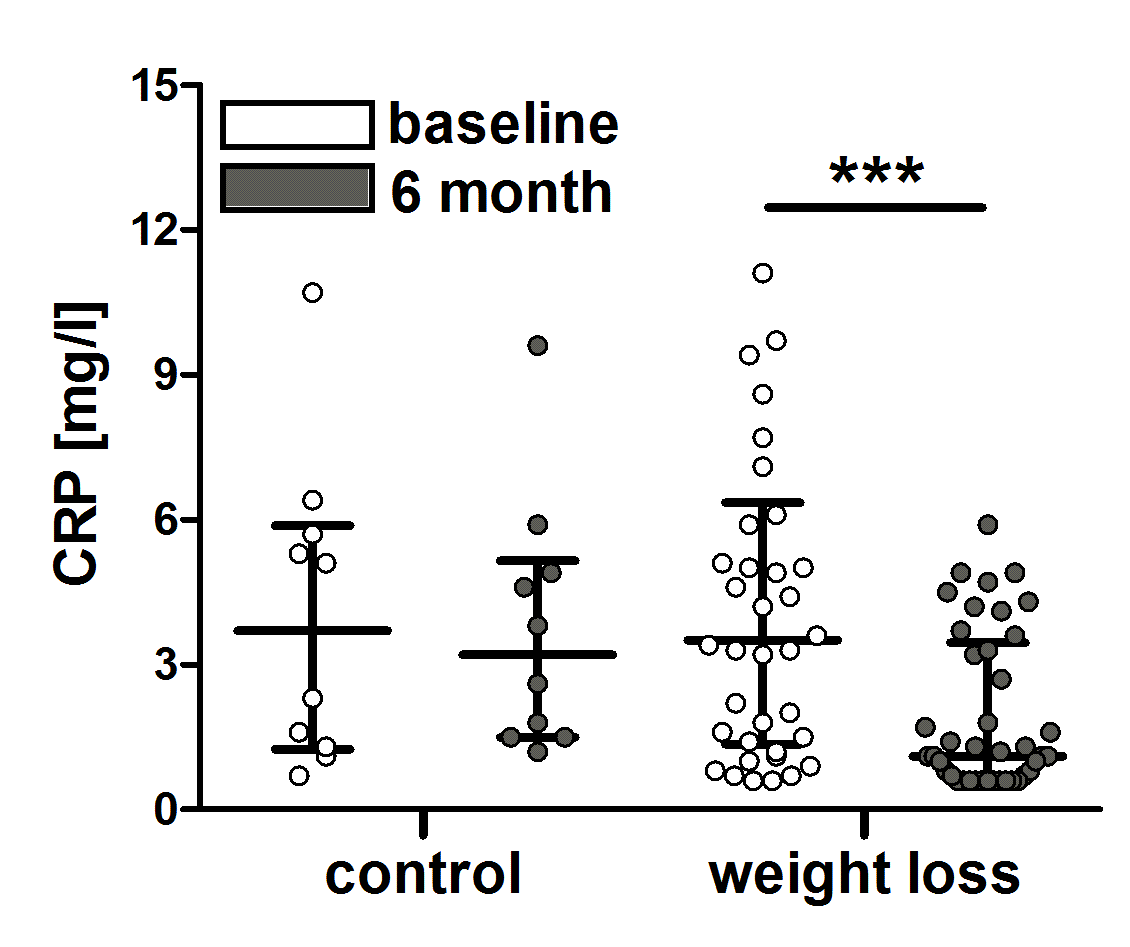


**Supplementary Figure 1 (a – d):** **Parameters for weight loss and CRP as an inflammation marker before and after the 6-month intervention period**. Subjects with MetS before and after the 6-month intervention period were analyzed. The Wilcoxon Signed-Rank test was used to analyze differences in paired samples of the treatment arm (N = 39) and the control arm (N = 12), **p* < 0.05, ***p* < 0.01, ****p* < 0.001. BMI= body mass index; hsCRP= high sensitive C-reactive protein; WBC= white blood cell count.


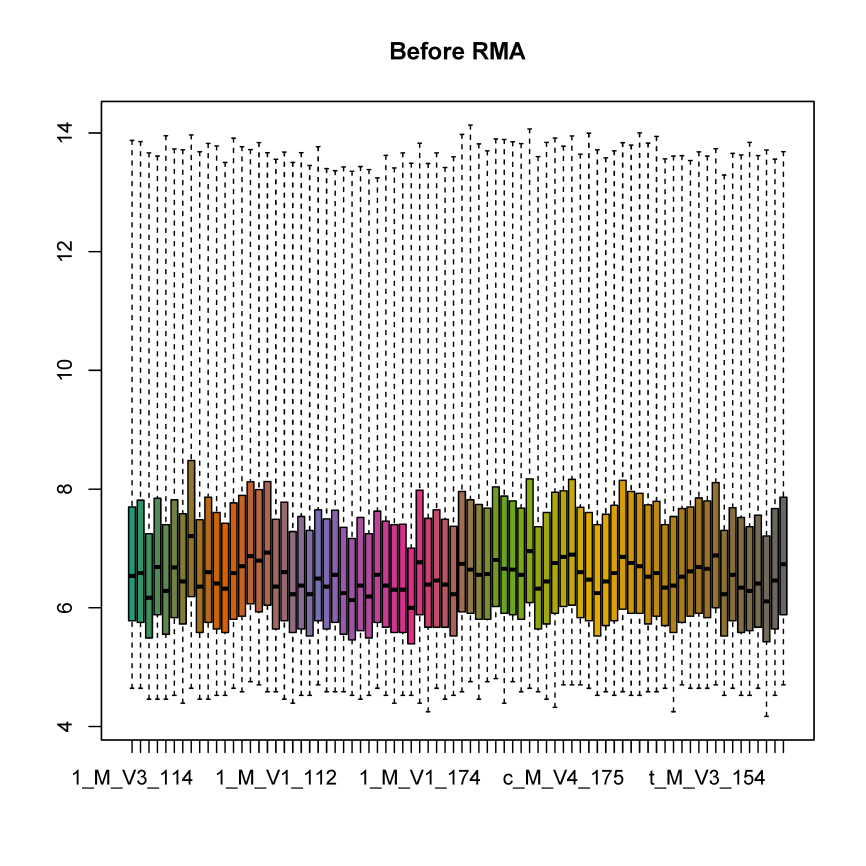

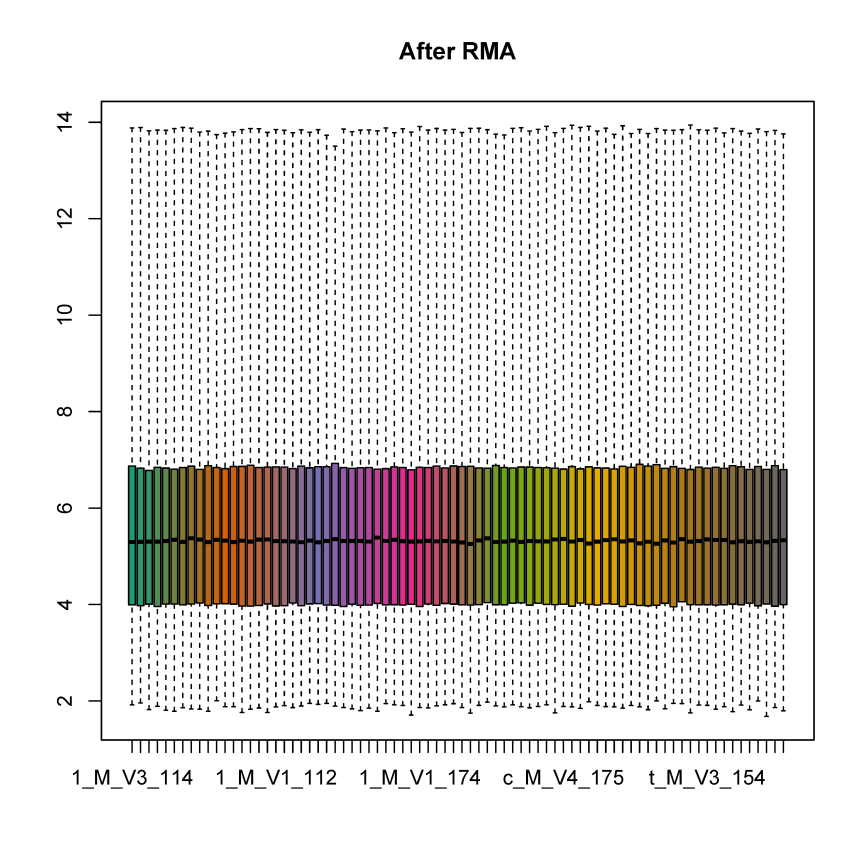


**Supplementary Figure 2: Data normalization.** Visualization of the gene expression data before and after robust multiarray average (RMA) normalization. The figure shows monocyte samples of the treated arm. RMA was generated using the affycoretools package in R; R package version 1.54.0.

**
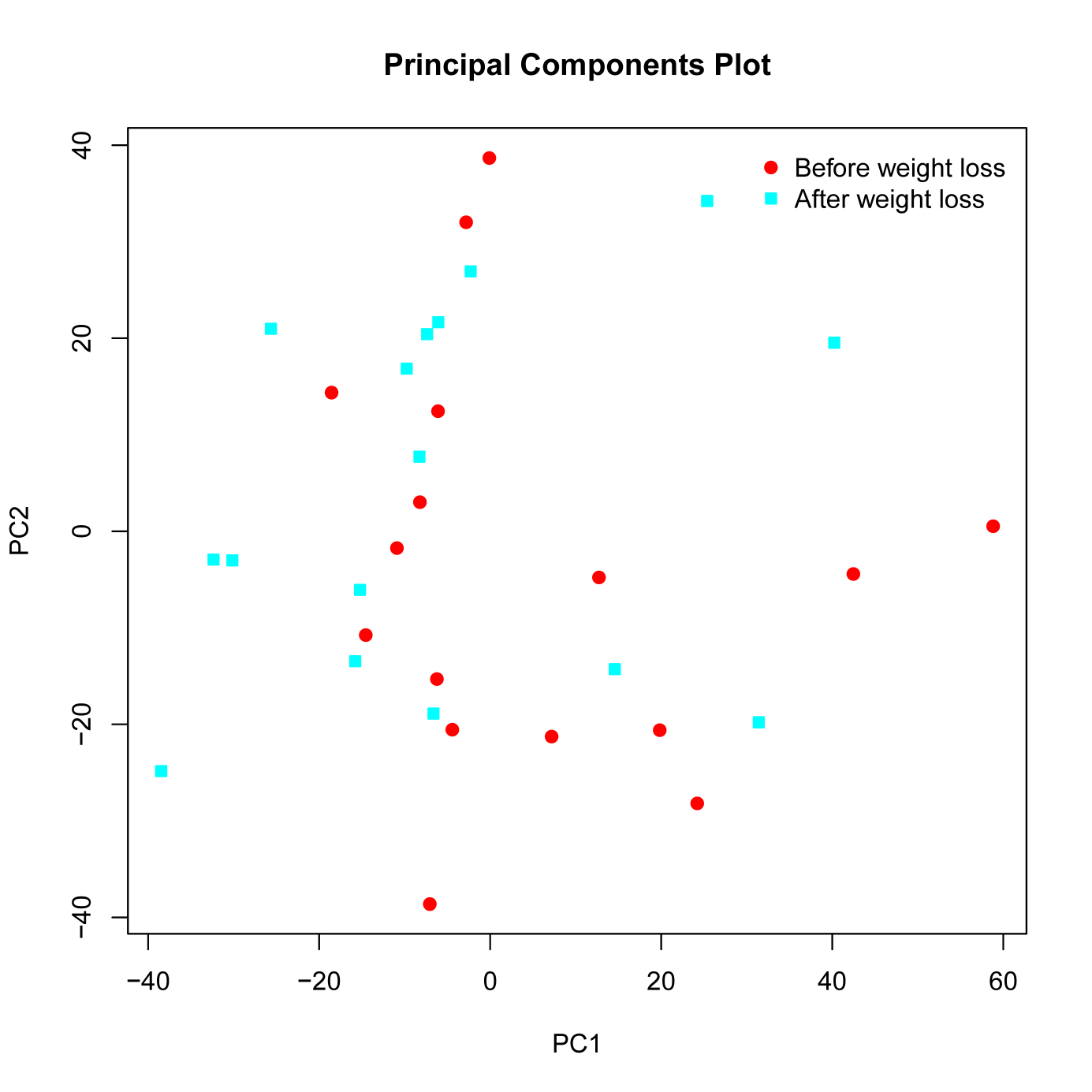
**

**Supplementary Figure 3:** The principal components analysis (PCA) plot was used to visualize the variance in the gene expression data in participants of the treatment arm in which elevated hsCRP levels were reduced by at least 50% following weight loss (N=17, hsCRP subgroup). The first principal component (PC1) on the x-axis is the linear combination comprising the largest percentage of variation in the dataset. The second principal component (PC2), located on the y-axis, captures the second largest percentage of variance. The profile before weight loss is shown in red, the profile after weight loss is shown in blue. PCA was generated using the affycoretools package in R (R package version 1.54.0).

| Triglycerides [%] | **.617**** |  |  |  |  |  |  |  |  |  |  |
| --- | --- | --- | --- | --- | --- | --- | --- | --- | --- | --- | --- |
| Total cholesterol [%] | **.372**** | **.395**** |  |  |  |  |  |  |  |  |  |
| HDL-cholesterol [%] | **-.471**** | **-.538**** | .226 |  |  |  |  |  |  |  |  |
| LDL-cholesterol [%] | .203 | -,049 | **.884**** | **.503**** |  |  |  |  |  |  |  |
| HOMA-IR [%] | **.613**** | **.510**** | .265 | **-.396**** | .036 |  |  |  |  |  |  |
| Total fat mass [%] | **.934**** | **.528**** | .226 | **-.526**** | .016 | **.633**** |  |  |  |  |  |
| Leptin [%] | **.635**** | **.280*** | .265 | -.224 | 0,325* | **.499**** | **.894**** |  |  |  |  |
| IL-6 [%] | .089 | .106 | -.051 | **-.284*** | -.152 | -.084 | .185 | .229 |  |  |  |
| hsCRP [%] | **.506**** | .070 | .100 | -.082 | .169 | **.322*** | **.743**** | **.553**** | **.294*** |  |  |
| WBC [%] | .236 | **.289*** | .027 | **-.375**** | -.109 | .276 | .150 | .175 | .072 | -.046 |  |
| CD14^+^ cell frequencies [%] | **.369**** | .072 | .162 | .110 | .173 | .166 | .343 | **.429**** | .163 | **.348*** | .074 |
|  | BMI [%] | Triglycerides [%] | Total cholesterol [%] | HDL-cholesterol [%] | LDL-cholesterol [%] | HOMA-IR [%] | Total fat mass [%] | Leptin [%] | IL-6 [%] | hsCRP [%] | WBC [%] |

**Supplementary Table 1: Correlation of clinical parameters with CD14^+^ cell frequencies.** Spearman-Rho correlation matrix (2-tailed) of percentual changes in anthropometric and metabolic characteristics of study participants of both arms. Statistically significant correlations are printed bold-faced (* p < 0.05; ** p < 0.01).

|  |  | **hsCRP subgroup** | |
| --- | --- | --- | --- |
|  |  | **before** | **6 month** |
| age | median | 49.0 |  |
|  | IQR | (46.0-52.0) |  |
| BMI | median | 35.0 | 29.5*** |
|  | IQR | (31.2-35.6) | (26.2-32.0) |
| Body weight [kg] | median | 111 | 93.4*** |
|  | IQR | (102-115) | (88.0-101) |
| Triglycerides [mmol/l] | median | 1.74 | 1.22** |
|  | IQR | (1.33-3.27) | (0.92-2.10) |
| total cholesterol [mmol/l] | median | 5.63 | 5.03 |
|  | IQR | (4.64-6.50) | (4.23-6.02) |
| HDL-cholesterol [mmol/l] | median | 1.20 | 1.46*** |
|  | IQR | (1.09-1.35) | (1.21-1.56) |
| LDL-cholesterol [mmol/l] | median | 3.18 | 3.04 |
|  | IQR | (2.44-4.02) | (2.46-3.80) |
| Free fatty acids [mmol/l] | median | 0.59 | 0.41* |
|  | IQR | (0.47-0.71) | (0.35-0.53) |
| HOMA-IR | median | 2.84 | 1.23*** |
|  | IQR | (2.13-3.61) | (1.00-2.42) |
| Total fat mass [kg] | median | 31.3 | 22.1** |
|  | IQR | (26.0-36.0) | (19.1-27.3) |
| Leptin [ng/ml] | median | 13.5 | 5.09*** |
|  | IQR | (9.57-20.3) | (2.39-7.86) |
| IL-6 [pg/ml] | median | 3.10 | 2.20** |
|  | IQR | (2.25-4.10) | (1.90-2.80) |
| hsCRP [mg/l] | median | 4.20 | 1.20*** |
|  | IQR | (3.25-10.4) | (0.90-1.65) |
| WBC [Gpt/l] | median | 6.20 | 6.40 |
|  | IQR | (5.70-7.85) | (5.30-7.55) |
| CD14^+^ cell frequencies [10^7^/20 ml] | median | 1.20 | 0.80** |
|  | IQR | (0.95-1.45) | (0.50-1.40) |

**Supplementary Table 2:** **Characteristics of subgroup analyses.** Clinical characteristics of participants of the treatment arm, who reduced elevated hsCRP levels by at least 50% following treatment (N=16, hsCRP subgroup). Data are presented as the median (interquartile range). The Wilcoxon Signed-Rank test was used to analyze differences in paired samples, **p* < 0.05, ***p* < 0.01, ****p* < 0.001. BMI= body mass index; HDL= high-density lipoprotein cholesterol; LDL= low-density lipoprotein cholesterol; HOMA-IR= homeostasis model of assessment index; IL-6= interleukin-6; hsCRP= high sensitive C-reactive protein; WBC= white blood cell count.

|  |  | **Initial treatment (N=22)** | | **Treated controls (N=17)** | |
| --- | --- | --- | --- | --- | --- |
|  |  | **before** | **6 month** | **before** | **6 month** |
| Age | median | 48.0 |  | 49.0 |  |
|  | IQR | (45.0-48.0) |  | (46.5-51.5) |  |
| BMI | median | 32.9 | 28.7*** | 34.7 | 28.8*** |
|  | IQR | (30.9-35.7) | (25.7-31.3) | (32.6-35.5) | (26.6-32.0) |
| Body weight [kg] | median | 111 | 95.5*** | 105 | 91.2*** |
|  | IQR | (98.5-119) | (85.5-106) | (99.0-115) | (82.7-102) |
| Triglycerides [mmol/l] | median | 1.90 | 1.27** | 1.90 | 1.02** |
|  | IQR | (1.27-3.91) | (1.01-1.90) | (1.42-2.72) | (0.86-1.72) |
| Total cholesterol [mmol/l] | median | 6.18 | 5.74* | 5.66 | 4.94 |
|  | IQR | (5.27-6.71) | (4.55-6.40) | (4.74-6.63) | (4.30-5.51) |
| HDL-cholesterol [mmol/l] | median | 1.21 | 1.33** | 1.27 | 1.48*** |
|  | IQR | (1.02-1.35) | (1.22-1.58) | (1.11-1.46) | (1.25-1.73) |
| LDL-cholesterol [mmol/l] | median | 3.69 | 3.65 | 3.00 | 2.78 |
|  | IQR | (3.38-4.63) | (2.34-4.14) | (2.30-4.14) | (2.45-3.31) |
| Free fatty acids [mmol/l] | median | 0.52 | 0.51 | 0.57 | 0.49* |
|  | IQR | (0.43-0.71) | (0.39-0.71) | (0.36-0.73) | (0.28-0.56) |
| HOMA-IR | median | 3.19 | 1.48** | 2.21 | 1.13** |
|  | IQR | (2.27-5.38) | (0.98-2.54) | (1.54-3.84) | (1.87-2.15) |
| Leptin [ng/ml] | median | 12.2 | 5.58*** | 13.5 | 5.57* |
|  | IQR | (7.49-16.62) | (2.94-8.30) | (8.76-19.25) | (2.04-9.52) |
| IL-6 [pg/ml] | median | 2.75 | 1.90 | 2.70 | 2.70 |
|  | IQR | (1.77-4.45) | (1.50-2.45) | (1.95-3.80) | (2.2-3.2) |
| hsCRP [mg/l] | median | 4.30 | 1.15*** | 3.20 | 1.10** |
|  | IQR | (1.70-8.37) | (0.77-2.82) | (0.85-5.40) | (0.60-4.25) |
| WBC [Gpt/l] | median | 5.75 | 6.10 | 7.10 | 6.20* |
|  | IQR | (4.82-7.02) | (4.70-7.00) | (5.95-7.95) | (4.80-7.60) |
| CD14^+^ cell frequencies [10^7^/20 ml] | median | 1.10 | 0.80 | 1.00 | 0.80** |
|  | IQR | (0.80-1.40) | (0.60-1.42) | (0.75-1.45) | (0.50-1.10) |

**Supplementary Table 3:** **Comparison of groups that were combined in the treatment arm.** Clinical characteristics of participants of the initial treatment arm (N=22) and participants of the control arm who participated in the subsequent 6-month weight loss program (N=17). No differences were found between both groups before weight loss (Mann-Whitney U test). The Wilcoxon Signed-Rank test was used to analyze differences in paired samples, **p* < 0.05, ***p* < 0.01, ****p* < 0.001. BMI= body mass index; HDL= high-density lipoprotein cholesterol; LDL= low-density lipoprotein cholesterol; HOMA-IR= homeostasis model of assessment index; IL-6= interleukin-6; hsCRP= high sensitive C-reactive protein; WBC= white blood cell count.
